# The Neuroprotective Effect of *Byu d Mar* 25 in LPS-Induced Alzheimer's Disease Mice Model

**DOI:** 10.1155/2021/8879014

**Published:** 2021-02-27

**Authors:** Lan Liu, Yongcang Zhang, Liang Tang, Hua Zhong, Dunzhu Danzeng, Cuiting Liang, Shanling Liu

**Affiliations:** ^1^Medical College, Tibet University, Lhasa, Tibet 850000, China; ^2^Department of Obstetrics & Gynecology, West China Second University Hospital, Sichuan University, Chengdu, Sichuan 610041, China; ^3^Key Laboratory of Birth Defects and Related Diseases of Woman and Children (Sichuan University), Ministry of Education, Sichuan 610041, China; ^4^Department of Human Anatomy, Histology and Embryology, Institute of Neuroscience, Changsha Medical University, Changsha 410219, China; ^5^Department of Anatomy, Chengdu University of Traditional Chinese Medicine, Chengdu, Sichuan 610041, China

## Abstract

Inflammatory factors play an important role in the pathogenesis of Alzheimer's disease (AD). *Byu d Mar* 25 (BM25) has been suggested to have protective effects in the central nervous system. However, the effect of BM25 on AD has not been determined. This study aims to investigate the neuroprotective effect of BM25 in AD. A total of 40 AD model mice were randomly assigned to the following five groups (*n* = 8 per group): the AD + NS group, the AD + donepezil group, and three AD + BM25 groups treated with either 58.39 mg/kg (AD + BM25-L), 116.77 mg/kg (AD + BM25-M), or 233.54 mg/kg BM25 (AD + BM25-H). The Morris water maze test was performed to assess alterations in spatial learning and memory deficits. Nissl staining was performed to detect Nissl bodies and neuronal damage. The expression of IL-1*β* and TNF-*α* was evaluated by ELISA. The protein expression of P-P38, P38, P-I*κ*B*α*, caspase 1, COX2, and iNOS was determined by western blotting. The expression of A*β*, p-Tau, and CD11b was measured by immunohistochemistry. The mRNA expression levels of IL-1*β*, TNF-*α*, COX2, and iNOS were measured by qRT-PCR. Spatial memory significantly improved in the AD + BM25-M and AD + BM25-H groups compared with the AD + NS group (*p* < 0.05). The expression of A*β* and p-Tau significantly decreased in the AD + BM25-M and AD + BM25-H groups (*p* < 0.05). The neuron density and hierarchy and number of pyramidal neurons significantly increased in the AD + BM25-M and AD + BM25-H groups (*p* < 0.05). In addition, the expression levels of CD11b, IL-1*β*, TNF-*α*, COX2, iNOS, caspase 1, p-I*κ*B*α*, and p-P38 significantly decreased in the AD + BM25-M and AD + BM25-H groups (*p* < 0.05). In conclusion, our findings suggest that BM25 may exert anti-inflammatory and neuroprotective effects in AD model mice by suppressing the activity of microglia and inhibiting the phosphorylation of I*κ*B*α* and p38 MAPK.

## 1. Introduction

Alzheimer's disease (AD) is a common neurodegenerative disease in the elderly population that causes declines in learning and memory [1–4]. The incidence of AD in people over the age of 65 is approximately 5% [5]. However, the pathogenesis of sporadic AD is still not fully understood. Neuroinflammation has been suggested to play an important role in the development of AD [6, 7]. At present, the role of glial cell activation, especially microglial cells, in neuroinflammation has been widely confirmed [8, 9].

*Byu d Mar* 25 (BM25) was developed by the Tibetan Medicine Master Dima Danzeng Peng Cuo in the 18th century and is still used today for multiple neurological disorders [10]. BM25 is composed of 25 rare herbs, such as saffron, calamus, and musk. It has the functions of opening the orifices and relieving pain. A clinical study has shown that BM25 has positive effects on neuropathic pain, epilepsy, stroke, and multiple peripheral neuropathies and neurological disorders [11]. In addition, BM25 has been shown to attenuate neuronal and astrocyte injury by inhibiting the neuronal denaturation and astrocyte overactivation induced by D-galactose [12]. These findings may indicate a potential therapeutic role for BM25 in AD.

The pharmacological mechanism of BM25 at the molecular level has been less studied. Du et al. found that BM25 reduced the expression levels of nitric oxide (NO) and nitric oxide synthase (NOS) in the plasma of migraine rat models [11]. Liu et al. reported that BM25 inhibited the phosphorylation of NF-kB P65 in human neuroblastoma cells (SH-SY5Y) [13]. The results of network pharmacological analysis suggested that the anti-AD mechanism of BM25 might be related to the regulation of the MAPK, insulin, and mTOR signal transduction pathways; intervention in inflammation and immunity; apoptosis and autophagy; and intervention in A*β* expression and clearance in brain tissue [14].

However, no systematic research on the effect and mechanism of BM25 in AD has been conducted. In the present study, we aimed to illustrate the effect and mechanism of BM25 in an LPS-induced AD mouse model.

## 2. Materials and Methods

### 2.1. Animals and Drug Administration

This experiment followed the ethical standards of the Declaration of Helsinki as well as national and international guidelines. The research procedures were approved by the Ethics Committee of Tibet University, China (EC20190018). The LPS-induced AD mouse model was established according to our previous study [15]. A total of 40 LPS-induced AD mice were randomly divided into the following five groups:

1. AD + donepezil group (donepezil (1 mg/ml), 0.1 ml/10 g) (*n* = 8)
2. AD + NS group (normal saline (NS) (0.9%), 0.1 ml/10 g) (*n* = 8)
3. AD + BM25-L group (low dose, L) (58.39 mg/kg, 0.1 ml/10 g) (*n* = 8)
4. AD + BM25-M group (medium dose, M) (116.77 mg/kg, 0.1 ml/10 g) (*n* = 8)
5. AD + BM25-H group (high dose, H) (233.54 mg/kg, 0.1 ml/10 g) (*n* = 8)

The dosing and duration of BM25 followed the studies conducted by Du et al.[16] and Li et al.[17]. Drug treatments were performed by lateral ventricular stereotactic injection and lasted for four weeks. The Morris water maze test was performed on the last day of treatment to assess the alterations in spatial learning and memory deficits. Nissl staining was performed to detect Nissl bodies and neuronal damage. The expression of IL-1*β* and TNF-*α* was evaluated by ELISA. The expression of P-P38, P38, P-I*κ*B*α*, Caspase1, COX2, and iNOS proteins was determined by western blotting. The expression of A*β*, p-Tau, and CD11b was measured by immunohistochemistry. The mRNA expression levels of IL-1*β*, TNF-*α*, COX2, and iNOS were measured by qRT-PCR.

### 2.2. Morris Water Maze Test

Spatial learning and memory deficits in the five groups were evaluated by the Morris water maze on the last day of treatment. The test protocols followed a previously published study by Vorhees et al.[18]. An ANY-maze Video Tracking System (Stoelting Co., USA) was used to track and record animal movement during the trials. The swim path, escape latency, and frequency of crossing the target platform were recorded and analyzed.

### 2.3. Tissue Collection

The mice were anesthetized with pentasorbital sodium (0.2%, 0.1 ml/10 g) by intraperitoneal injection. The brain tissue samples (*n* = 8) from each group were stored in 10% neutral formalin, and other specimens (*n* = 8) were stored at −80°C until further analysis.

### 2.4. Nissl Staining

Nissl bodies in the cytoplasm of surviving neurons were detected by Nissl staining (Beyotime Institute of Biotechnology, China). The number of positive cells per unit area (mm^2^) at the same site in the hippocampus was detected by using Image-Pro Plus 5.1 software (Media Cybernetics, Inc., Bethesda).

### 2.5. Enzyme-Linked Immunosorbent Assay (ELISA)

The expression levels of IL-1*β* and TNF-*α* in brain tissues were measured by ELISA with ELISA kits that were purchased from Sigma (Tokyo, Japan). The levels of IL-1*β* and TNF-*α* were detected by a microplate spectrophotometer (Multiskan MK, Finland). The measurement data are expressed as the mean ± standard deviation (SD).

### 2.6. Western Blotting

The protein expression levels of p-p38, p38, p-I*κ*B*α*, Caspase1, COX2, and iNOS were detected by western blotting. The total protein concentration of the brain tissues was analyzed with a BCA kit (Sigma, CA, USA). The blots were separately probed with rabbit antibodies against p-p38 (1 : 1000; 43 kDa, Affinity Biosciences), p38 (1 : 3000; 43 kDa, Affinity Biosciences), p-I*κ*B*α* (1 : 1000; 39 kDa, Affinity Biosciences), Caspase1 (1 : 1000; 45 kDa, Affinity Biosciences), COX2 (1 : 1000; 72 kDa, Affinity Biosciences), iNOS (1 : 500; 130 kDa, Affinity Biosciences), and *α*-Tubulin (1 : 5000; ProMab). Subsequently, the blots were probed with horseradish peroxidase- (HRP-) conjugated goat secondary antibody against rabbit IgG (1 : 80000; Affinity Biosciences). Quantitative analysis of the protein bands was performed with Image-Pro Plus 5.1 software (Media Cybernetics, Inc., Bethesda).

### 2.7. Immunohistochemistry

The tissues were thoroughly rinsed with PBS, treated with 3% H_2_O_2_ for 20 min, and incubated with 5% horse serum at room temperature for 1 h. Then, the tissues were separately incubated with mouse antibody against A*β*1-40 (1 : 150; Affinity Biosciences), p-Tau (1 : 200; Affinity Biosciences), or CD11b (1 : 150; Affinity Biosciences) at 4°C overnight and reacted with biotinylated broad-spectrum antibody against rabbit IgG (1 : 200; Affinity Biosciences) at room temperature for 2 h. Finally, the immunoreactive product was visualized by incubation in 0.05% DAB (Affinity Biosciences) for 3 min. The absorbance was analyzed by Image-Pro Plus 5.1 software (Media Cybernetics, Inc., Bethesda).

### 2.8. qRT-PCR

TRIzol Reagent (Invitrogen, Grand Island, NY, USA) was used to isolate total RNA from each brain tissue sample. The RNA quantity and integrity were measured by an ultraviolet spectrophotometer (UV-9000) (Shanghai Precision Instrument Co., Ltd.). Total RNA samples were purified with DNase, and cDNA was synthesized by a SuperScript VILO™ cDNA kit (Thermo Fisher Scientific, NY, USA). qRT-PCR was performed using HieffTM qPCR SYBR® Green Master Mix (Takara Bio Inc., Dalian, China) on a LightCycler® 2.0. The Ct values were analyzed with SDS 2.0 software (PE Biosystems). The relative mRNA expression levels of IL-1*β*, TNF-*α*, COX2, and iNOS were analyzed using the 2^−ΔΔCt^ method and normalized to *β*-actin.

### 2.9. Statistical Analysis

All the data are presented as the mean ± standard deviation (mean ± SD). The significance of difference was analyzed by SPSS 22.0 followed by a *t*-test. A value of *p* < 0.05 was considered statistically significant.

## 3. Results

### 3.1. BM25 Significantly Decreased the Expression of A*β* and p-Tau and Improved Spatial Memory in the LPS-Induced AD Mouse Model

#### 3.1.1. Results of the Morris Water Maze

**Figure 1:**
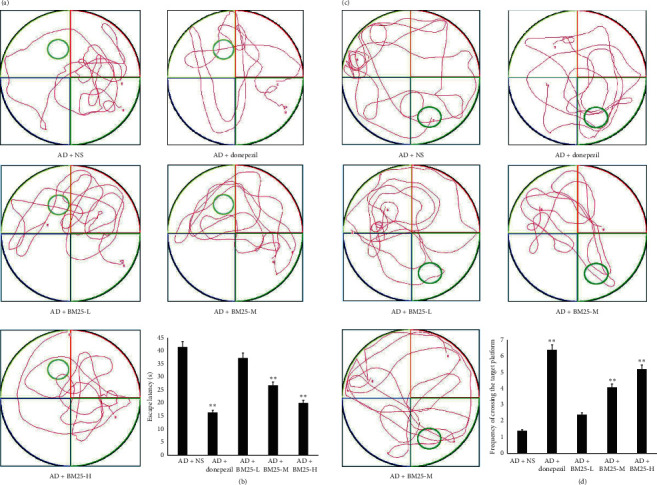
Testing of spatial learning and memory in AD + NS, AD + donepezil, and AD + BM25 groups by Morris water maze. (a) The swimming trajectory of mice in the AD + NS, AD + donepezil, and AD + BM25 groups. (b) Escape latency in AD + NS, AD + donepezil, and AD + BM25 groups. (c) Probe trial; the travel trajectory of mice in the AD + NS, AD + donepezil, and AD + BM25 groups. (d) The frequency of crossing the target platform in AD + NS, AD + donepezil, and AD + BM25 groups. A significant difference in escape latency in the AD+BM25 group rather than that in the AD+NS group was detected (^*∗∗*^*p* < 0.05). A more significant frequency in crossing the target platform in AD+BM25 groups than that in the AD+NS group was detected (^*∗∗*^*p* < 0.05). Data are expressed as the mean ± standard error of the mean (SEM). (*n* = 8/group in the AD + BM25 group; *n* = 8/group in the AD + donepezil group; *n* = 8/group in the AD + NS group). BM25: *Byu d Mar* 25; AD: Alzheimer's disease; NS: normal saline.

As shown in Figure 1, the mice in the AD + BM25-H and AD + BM25-M groups had shorter latencies and swimming distances to escape than the mice in the AD + NS group on the visible platform tests, indicating stronger spatial learning ability in AD mice treated with BM25 (*p* < 0.05). In the probe trial, the mice in the AD + BM25-H and AD + BM25-M groups spent significantly less time traveling into the fourth quadrant, where the hidden platform was previously placed, than the mice in the AD + NS group, which revealed better spatial memory ability in the AD mice treated with BM25 (*p* < 0.05).

#### 3.1.2. Expression of *Aβ and P-Tau*

**Figure 2:**
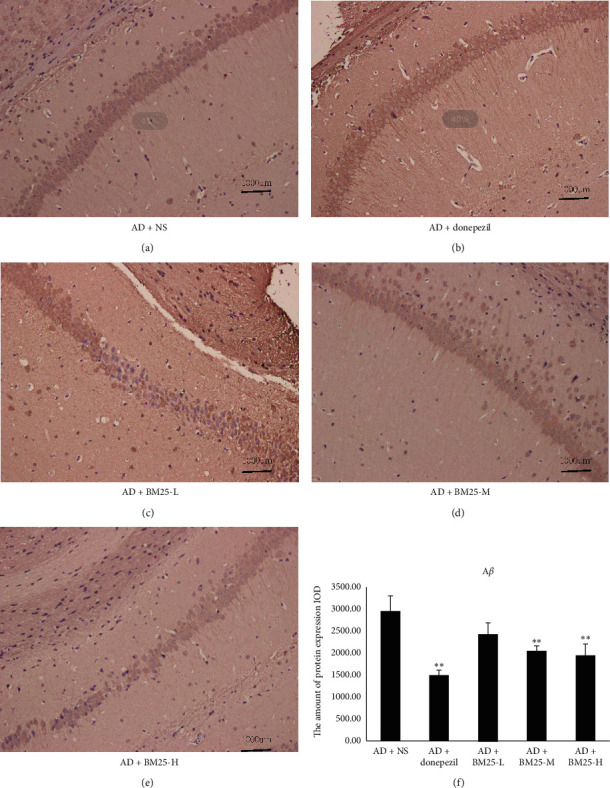
Light microscopic images show the distribution of A*β* immunolabeling across the brain of AD + NS, AD + donepezil, and AD + BM25 groups. (a) The distribution of A*β* immunolabeling in the AD + NS group. (b) The distribution of A*β* immunolabeling in the AD + donepezil group. (c) The distribution of A*β* immunolabeling in AD + BM25-L. (d) The distribution of A*β* immunolabeling in AD + BM25-M. (e) The distribution of A*β* immunolabeling in AD + BM25-H. (f) The comparison of A*β* in AD + NS, AD + donepezil, and AD + BM25 groups. The images revealed that the A*β* was highly expressed in the AD + NS group compared with the AD + BM25 groups. Data are expressed as the mean ± standard error of the mean (SEM) (*n* = 8/group in the AD + BM25 group; *n* = 8/group in the AD + donepezil group; *n* = 8/group in the AD + NS group). BM25: *Byu d Mar* 25; AD: Alzheimer disease; NS: normal saline.

**Figure 3:**
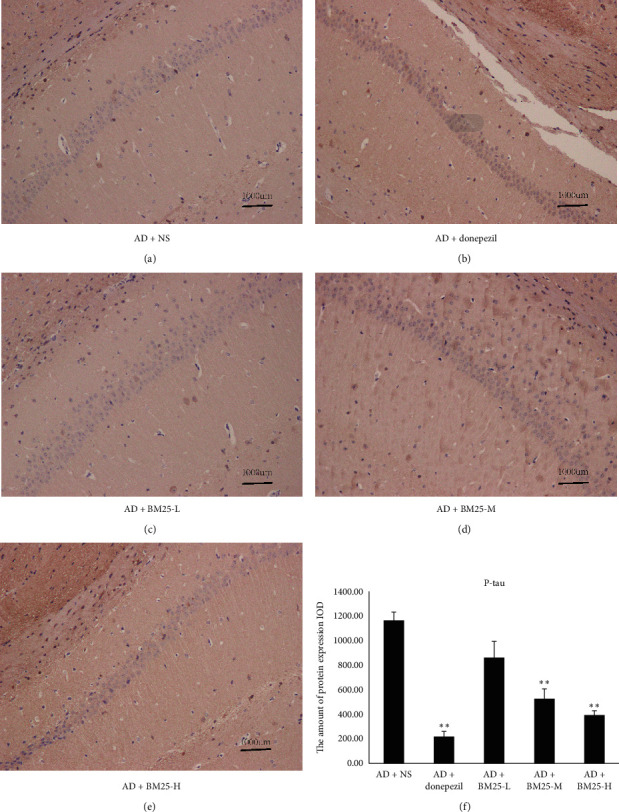
Light microscopic images show the distribution of p-Tau immunolabeling across the brain of AD + NS, AD + donepezil, and AD + BM25 groups. (a) The distribution of p-Tau immunolabeling in the AD + NS group. (b) The distribution of p-Tau immunolabeling in the AD + donepezil group. (c) The distribution of p-Tau immunolabeling in AD + BM25-L group. (d) The distribution of p-Tau immunolabeling in AD + BM25-M. (e) The distribution of p-Tau immunolabeling in AD + BM25-H. (f) The comparison of p-Tau in AD + NS, AD + donepezil, and AD + BM25 groups. The images revealed that the p-Tau was highly expressed in the AD + NS group compared with the AD + BM25 groups. Data are expressed as the mean ± standard error of the mean (SEM) (*n* = 8/group in the AD + BM25 group; *n* = 8/group in the AD + donepezil group; *n* = 8/group in the AD + NS group). BM25: *Byu d Mar* 25; AD: Alzheimer's disease; NS: normal saline.

Figures 2 and 3 show that the expression of A*β* and p-Tau was significantly lower in the AD + BM25-H (A*β*: 1937.75 ± 264.35; p-Tau: 394.87 ± 36.26) and AD + BM25-M groups (A*β*: 2040.46 ± 116.74; p-Tau: 529.98 ± 78.53) than in the AD + NS group.

### 3.2. BM25 Reduced Neuronal Damage and Neuronal Loss

#### 3.2.1. Nissl Staining

**Figure 4:**
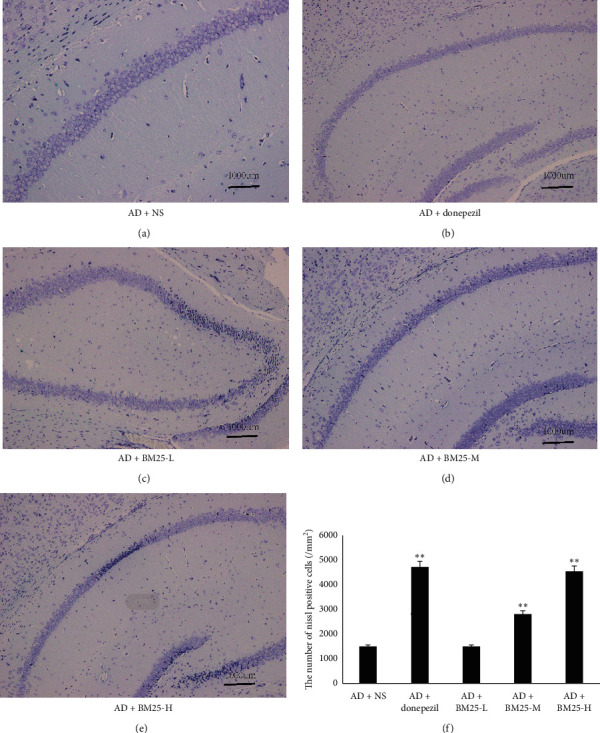
The results of Nissl staining. (a) Nissl staining in the AD + NS group. (b) Nissl staining in the AD + donepezil group. (c) Nissl staining in the AD + BM25-L group. (d) Nissl staining in the AD + BM25-M group. (e) Nissl staining in the AD + BM25-H group. (f) The comparison of the number of pyramidal neurons in AD + NS, AD + donepezil, and AD + BM25 groups. A significant difference of the neuron density and hierarchy and number of pyramidal neurons was observed between the AD + BM25 and AD + NS groups. Data are expressed as the mean ± standard error of the mean (SEM) (*n* = 8/group in AD + BM25 group; *n* = 8/group in the AD + donepezil group; *n* = 8/group in the AD + NS group). BM25: *Byu d Mar* 25; AD: Alzheimer's disease; NS: normal saline.

Under a light microscope, the pyramidal neurons in the hippocampal area of the AD + BM25-M and AD + BM25-H groups were arranged in a regular order with light staining of nuclei and clear staining of the cytoplasm in the AD + BM25-M and AD + BM25-H groups. In contrast, the neuron density and hierarchy decreased, the number of pyramidal neurons decreased, the neuron arrangement was disordered, the cell spacing increased, and neurons were significantly lost in the AD + NS group (Figure 4).

#### 3.2.2. Expression of the Caspase 1 Protein

**Figure 5:**
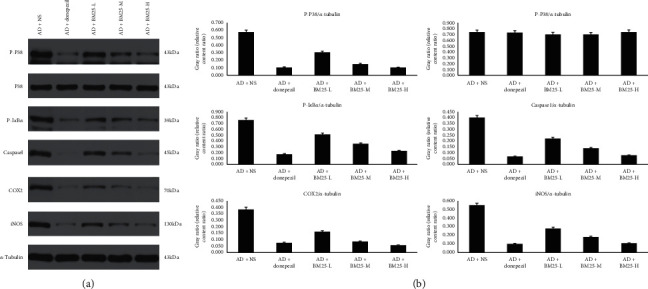
The expression levels of p-P38, P38, p-I*κ*B*α*, caspase 1, COX2, and iNOS proteins in AD + NS, AD + donepezil, and AD + BM25 groups. Quantitative summaries of the protein levels relative to *α*-tubulin as an internal control, expressed as a percentage of *α*-tubulin optical density (o.d.) for the groups (*n* = 8/group). Statistical results (Kruskal–Wallis nonparametric test with Dunn's multiple post-hoc comparison) were shown in the bar graphs, with “^∗∗^” indicating significant intergroup difference. Data are expressed as the mean ± standard error of the mean (SEM) (*n* = 8/group). (a) Expression levels of p-P38 in AD + NS, AD + donepezil, and AD + BM25 groups; (b) expression levels of P38 in AD + NS, AD + donepezil, and AD + BM25 groups; (c) expression levels of p-I*κ*B*α* in AD + NS, AD + donepezil, and AD + BM25 groups; (d) expression levels of Caspase1 in AD + NS, AD + donepezil, and AD + BM25 groups; (e) expression levels of COX2 in AD + NS, AD + donepezil, and AD + BM25 groups; (f) expression levels of iNOS in AD + NS, AD + donepezil, and AD + BM25 groups. BM25: *Byu d Mar* 25; AD: Alzheimer's disease; NS: normal saline.

As shown in Figure 5, the relative expression of the caspase 1 protein was significantly lower in the AD + BM25-H (0.081 ± 0.024) and AD + BM25-M groups (0.140 ± 0.014) than in the AD + NS group (0.400 ± 0.102) (*p* < 0.05).

### 3.3. BM25 Inhibited the Activity of Microglia and Decreased the Expression Levels of IL-1*β*, TNF-*α*, COX2, and iNOS

#### 3.3.1. Activity of Microglia

**Figure 6:**
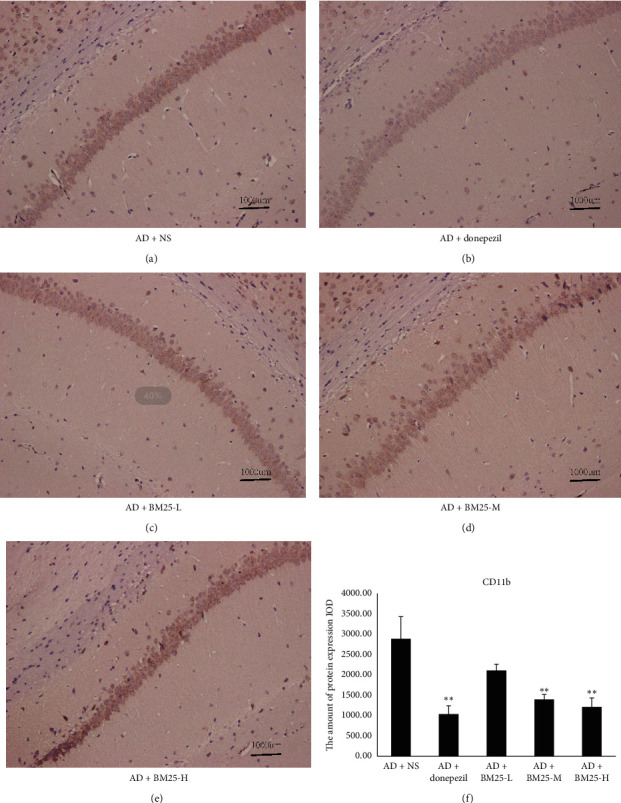
Light microscopic images show the distribution of CD11b immunolabeling across the brain of AD + NS, AD + donepezil, and AD + BM25 groups. (a) The distribution of CD11b immunolabeling in the AD + NS group. (b) The distribution of CD11b immunolabeling in the AD + donepezil group. (c) The distribution of CD11b immunolabeling in AD + BM25-L group. (d) The distribution of CD11b immunolabeling in AD + BM25-M. (e) The distribution of CD11b immunolabeling in AD + BM25-H. (f) The comparison of p-Tau in AD + NS, AD + donepezil, and AD + BM25 groups. The images revealed that the CD11b was highly expressed in the AD + NS group compared with AD + BM25 groups. Data are expressed as the mean ± standard error of the mean (SEM) (*n* = 8/group in the AD + BM25 group; *n* = 8/group in the AD + donepezil group; *n* = 8/group in the AD + NS group). BM25: *Byu d Mar* 25; AD: Alzheimer's disease; NS: normal saline.

As shown in Figure 6, the microglial cells in the hippocampus of the AD + BM25-H and AD + BM25-M groups were small, rod-shaped, and thin and had few branches. The microglial cells in the AD + NS group were branched, and the cell bodies became larger and rounder with more branches. The number of activated microglial cells (CD11b-positive cells) in the AD + NS group was significantly increased compared with those in the AD + BM25-H (1216.63 ± 217.91) and AD + BM25-M groups (1404.20 ± 120.01) (*p* < 0.05).

#### 3.3.2. Expression of Proinflammatory Cytokines

**Figure 7:**
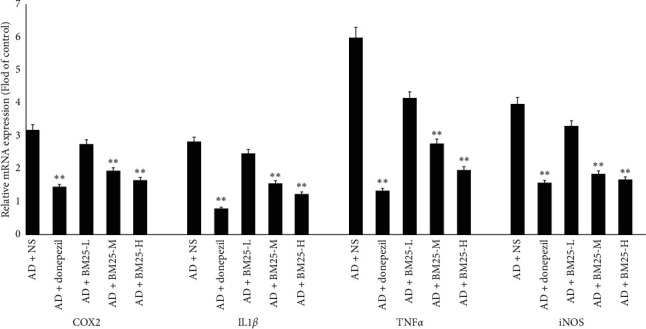
The expression levels of IL-1*β*, TNF-*α*, COX2, and iNOS mRNAs in brain tissues in AD + NS, AD + donepezil, and AD + BM25 groups. Data are expressed as the mean ± standard error of the mean (SEM) (*n* = 8/group). “^∗∗^” indicates a significant intergroup difference. BM25: *Byu d Mar* 25; AD: Alzheimer's disease; NS: normal saline.

**Figure 8:**
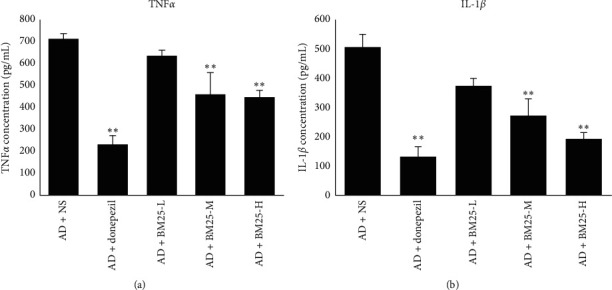
The expression levels of IL-1*β* and TNF-*α* in AD + NS, AD + donepezil, and AD + BM25 groups. (a) IL-1*β*; (b) TNF-*α*. Data are expressed as the mean ± standard error of the mean (SEM) (*n* = 8/group). “^∗∗^” indicates a significant intergroup difference. BM25: *Byu d Mar* 25; AD: Alzheimer's disease; NS: normal saline.

The relative mRNA expression levels of IL-1*β*, TNF-*α*, COX2, and iNOS in the AD + BM25-H and AD + BM25-M groups were found to be significantly lower than those in the AD + NS group (*p* < 0.05) (Figure 7). The ELISA results showed that the protein expression of IL-1*β* and TNF-*α* in the brain was significantly decreased in the AD + BM25-H (IL-1*β*: 192.64 ± 22.49; TNF-*α*: 445.58 ± 33.73) and AD + BM25-M (IL-1*β*: 274.22 ± 56.87; TNF-*α*: 461.18 ± 100.14) groups compared with the AD + NS group (IL-1*β*: 505.69 ± 43.33; TNF-*α*: 714.65 ± 23.00) (*p* < 0.05) (Figure 8). In addition, western blotting revealed that the number of COX2-and iNOS-positive dots (relative content ratio) in the AD + BM25-M (COX2: 0.096 ± 0.019; iNOS: 0.182 ± 0.020) and AD + BM25-H groups (COX2: 0.063 ± 0.025; iNOS: 0.108 ± 0.011) was significantly decreased compared with that in the AD + NS group (COX2: 0.375 ± 0.014; iNOS: 0.548 ± 0.126) (*p* < 0.05) (Figure 5).

### 3.4. BM25 Suppressed the Phosphorylation of I*κ*B*α* and p38 MAPK

Western blotting showed that the relative expression levels of p-I*κ*B*α* and p-P38 MAPK were significantly lower in the AD + BM25-M (p-I*κ*B*α*: 0.353 ± 0.012; p-p38 : 0.152 ± 0.022) and AD + BM25-H groups (p-I*κ*B*α*: 0.229 ± 0.015; p-p38 : 0.109 ± 0.019) than in the AD + NS group (p-I*κ*B*α*: 0.758 ± 0.021; p-p38 : 0.577 ± 0.024) (*p* < 0.05). Thus, BM25 can inhibit the phosphorylation of I*κ*B*α* and p38 MAPK (Figure 5).

## 4. Discussion

The etiology of AD is complex. Neuroinflammation is one of the main factors involved in the occurrence and development of AD and one of the important therapeutic targets for AD [19]. At present, the role of glial cells, especially microglia, in neuroinflammation has become a hotspot of research. Studies have shown that the inflammatory response induced by microglial activation is one of the pathogeneses of AD [20, 21]. Microglia can be activated and then produce a large number of proinflammatory factors, such as interleukin-1*β* (IL-1*β*) and tumor necrosis factor-*α* (TNF-*α*) [22]. Research has shown that IL-1*β* can be induced by the beta amyloid precursor (beta amyloid, *Aβ*) and cause *Aβ* deposition and Tau protein phosphorylation by reducing the expression of genes related to *Aβ* clearance [23]. Additionally, *Aβ* can bind to a specific receptor, activate microglia, and promote the release a large number of inflammatory factors and toxic substances [24]. Furthermore, activated microglial cells can express large quantities of iNOS and produce excess NO, which damages neurons by inhibiting cytochrome oxidase in the mitochondria of neurons [25]. Cox-2 is an inducible isoenzyme that is expressed in small amounts in microglia at rest. Under the action of proinflammatory molecules such as LPS, intracellular COX-2 mRNA levels increase, microglia are activated, and inflammatory mediators such as TNF-*α* and IL-6 are released [26].

BM25 is mainly used to invigorate the circulation of blood and to remove blood stasis. Chen et al. showed that calamus reduced the expression of the aquaporin-4 gene in glial cells [27]. Shi et al. reported that musk extract had a significant protective effect against the inflammatory damage of nerve cells caused by LPS, possibly by reducing the secretion of IL-6 by glial cells [28]. Our results confirmed that BM25 can significantly reduce the production of TNF-*α*, IL-1*β*, iNOS, COX-2, *Aβ*, and p-Tau and improve spatial memory, suggesting that BM25 may improve AD-like symptoms by inhibiting the activation of microglia, reducing the expression of proinflammatory cytokines and altering A*β* and p-Tau expression and clearance in brain tissue.

The NF-*κ*B pathway plays an important role in LPS-induced microglia [29]. Liu et al. showed that LPS activates the NF-*κ*B signaling pathway in microglia by binding to TLR4 and activating the expression of chemokines, proinflammatory cytokines and other genes [30]. The I*κ*B family includes I*κ*B*α*, I*κ*B*β*, and I*κ*B*ε*. I*κ*B*α* is the most important inhibitor of NF-*κ*B. When stimulated by an external signal, the I*κ*B kinase (IKK) complex is activated. Activated IKK phosphorylates IKK*α* and IKK*β*, which subsequently bind to ubiquitin ligases. I*κ*B*α* is ubiquitinated and degraded by the proteasome, leading to NF-*κ*B activation [31, 32]. Thus, the phosphorylation of I*κ*B is essential for NF-*κ*B activation. Currently, NF-*κ*B target genes include cytokines and inflammatory mediators (such as TNF-*α*, IL-6, IL-1, and iNOS). Excessive activation of NF-*κ*B leads to the production of a large number of inflammatory cytokines, which aggravates the inflammatory response. In the present study, the phosphorylation of I*κ*B*α* was detected by western blotting, and the results showed that BM25 significantly reduced the phosphorylation of I*κ*B*α*, which indicated that BM25 may act as an anti-inflammatory agent by suppressing the phosphorylation of I*κ*B*α* and inhibiting the expression of cytokines and inflammatory mediators (such as TNF-*α*, IL-1*β*, iNOS, and COX-2).

In addition to the NF-*κ*B signaling pathway, MAPK signaling plays an important role in the expression of inflammation-related factors after microglial activation. Youssef et al. showed that LPS can quickly activate p38, ERK, and JNK signaling in microglia [33]. Other molecules, such as ATP, thrombin, and TNF-*α*, also activate the MAPK signaling pathway, causing microglial activation [34]. Mitogen-activated protein kinases (MAPKs) are a type of serine/threonine protein kinase in cells [35]. Normally, MAPK exists in cells in a nonphosphorylated form. Stimulation via recognition of LPS by TLR4 receptors on the surface of microglia can induce the phosphorylation of MAPK and activate the expression of related cytokines and inflammatory mediator genes [36]. Researchers have suggested that, among the three MAPK subfamilies (p38 MAPK, JNK, and ERK), p38 MAPK is most closely related to the inflammatory response [37]. Studies have shown that LPS promotes the phosphorylation of P38 MAPK in a dose-dependent and time-dependent manner, thereby promoting the expression of inflammatory mediators such as TNF-*α*, IL-1*β*, and iNOS. Inhibiting the activation of P38 MAPK can inhibit the production of inflammatory mediators and protect neurons [38–40]. In the present study, the phosphorylation of P38 MAPK was detected by western blotting, and the results showed that BM25 significantly reduced the phosphorylation of P38 MAPK. Therefore, the results of this study suggest that BM25 may inhibit the release of inflammatory mediators by inhibiting the P38 MAPK pathway.

However, this study only investigated the effects and preliminary molecular mechanism of inflammatory factor release in an LPS-induced AD mouse model treated with BM25. Cell culture experiments are still lacking at present. Therefore, it will be necessary to carry out cell experiments to better explain the anti-inflammatory and neuroprotective effects of BM25 in neuronal cells.

## 5. Conclusion

BM25 can significantly improve spatial memory, reduce neuronal apoptosis and death, and inhibit the production of A*β*, p-Tau, IL-1*β*, iNOS, COX-2, and TNF-*α* in an LPS-induced AD mouse model. Furthermore, BM25 can exert anti-inflammatory and neuroprotective effects by inhibiting the phosphorylation of I*κ*B*α* in the NF-*κ*B signaling pathway and p38 MAPK in the MAPK signaling pathway.

## Data Availability

The data used to support the findings of this study are included within the article.
